# Letter to the editor: Measles cases among fully vaccinated persons

**DOI:** 10.2807/1560-7917.ES.2018.23.34.1800449

**Published:** 2018-08-23

**Authors:** Yuzo Arima, Kazunori Oishi

**Affiliations:** 1 Infectious Disease Surveillance Center, National Institute of Infectious Diseases, Tokyo, Japan

**Keywords:** measles, vaccination coverage, immunization, epidemiology

**To the editor:** We read with great interest the rapid communication by Bernadou et al. [1] regarding the measles outbreak in France. In particular, we noted that the proportion of measles cases who were fully vaccinated (i.e. with two doses of measles-mumps-rubella-containing vaccine) was higher (14 %) than what was observed in France during the much larger epidemic that occurred between 2008 and 2011 (3–4 %). As the authors state, rising proportions of vaccinated cases have been observed recently in several countries [2-4].

Importantly, increasing proportions of vaccinated measles cases can be interpreted by the public or the media, incorrectly, as an indication of an ineffective vaccine. We appreciate that the authors state that further investigation is needed, and we would also like to comment on this ‘paradox’.

While measles-containing vaccine (MCV) is highly effective, a small proportion of vaccinated persons (i.e. 3 % [5]) exposed to the virus are known to manifest as measles despite having been vaccinated with two doses of MCV. With increased vaccination coverage in the general population, the number of fully vaccinated individuals increases, and proportionate to this increase, the frequency of fully vaccinated measles cases would be expected to increase (since a small percent of the fully vaccinated are known to contract measles), as long as the measles virus circulates. Moreover, as the number of unvaccinated individuals remaining in the population decreases, in proportion to this decline, the frequency of unvaccinated measles cases is expected to decrease (assuming that all other factors remain unchanged; herd protection could further decrease the likelihood of exposure to a measles case for the few unvaccinated persons). Thus, even with a highly effective vaccine, proportionate to the increase in the vaccinated and decrease in the unvaccinated, the proportion of fully vaccinated cases would, on average, be expected to increase (here, we are not considering cases imported from endemic areas or population-mixing dynamics).

However, as the absolute risk of measles is very low among those fully vaccinated, with increased vaccination coverage, we would expect a decrease in the incidence of cases in the general population. Even if the relative risk of measles in the fully vaccinated vs unvaccinated were constant, the increased weight of the fully vaccinated in the population would decrease the overall incidence. Compared with the considerably larger epidemic that occurred between 2008 and 2011 in France [6], we wonder if the increase in the proportion of fully vaccinated cases is reflecting the increased vaccination coverage [4], given the smaller size of the current outbreak in France [7,8]. As the authors comment, further investigations are warranted, and analyses by birth cohort or specific population groups may be informative—for instance, for subpopulations where vaccination coverage is high, the proportion of fully vaccinated cases may be high, but the incidence of measles low.

Any interpretation of risk needs to consider denominators. In considering the proportion of cases fully vaccinated—which is only a proportion of vaccination status among the numerator of cases—we need to be mindful of the number of vaccinated and unvaccinated individuals in the underlying population. As an extreme hypothetical example, if 100 % of the population were vaccinated twice, we might expect only about 3 % of those to become cases, but we would see that all the cases were fully vaccinated. With increasing vaccination coverage, the visibility of vaccinated cases will increase, and requires careful interpretation.

Thus, although the phenomenon of increasing proportion of fully vaccinated cases with increased vaccine coverage may seem counterintuitive [4], in the context of decreased incidence, this is expected. While waning immunity and increased sensitivity of investigations (i.e. detecting milder measles cases who are more likely to have been vaccinated [2,5]) may also explain some of the increased proportion of vaccinated cases, even in the absence of such conditions, this phenomenon would not be surprising. We commend Bernadou et al. for their rapid communication and add a brief epidemiological commentary, lest some in the media/public erroneously conclude that measles vaccine is not effective. Such misinterpretation can have negative implications for vaccine uptake, and reminds us of the importance of risk communication in public health.
